# Side effects and prescription errors of ocular drugs

**DOI:** 10.4103/0974-620X.71915

**Published:** 2010

**Authors:** Saqib Ali Khan Utman, Astrid Specht, Hanna Masud Baig

**Affiliations:** Bradford Royal Infirmary, Duckworth Lane, Bradford and Furness General Hospital, Abbey Way, Barrow-in-Furness, UK

Sir,

Drug prescription errors are common and 20% of all medical negligence treatment claims arise from incorrect use of prescription drugs.[1] Mistakes not only cost the individuals but also have a financial impact on the health services.[2] These errors can occur at several stages like, prescribing, transcription, dispensing and administration, side effects varying in severity from minimal, and thereby unrecognized, to fatal.[3,4]

We conducted an audit to determine the most frequently occurring side effects of the ocular drugs and severity of these effects and to ascertain preventable prescription errors. In our study, we randomly selected 31 prospective patients over the period of two months from July 2004 to Sept 2004. These patients presented in the out-patient clinic with prescription errors or side effects due to ocular medication. Details were recorded on the proformas during clinic consultations. Patients consented orally to participate in the audit, and were inducted during their normal routine follow up. Patients presenting with acute side effects of the ocular medicines were seen in the emergency clinic as per standard protocol already in place within the department and were followed up as per standard departmental protocol if required.

In our study, 20 (64.5%) patients had prescription errors and 11 (35.5%) experienced side effects due to ocular medications. Ophthalmologists were involved in ten (50%) prescription errors. These errors varied from prescribing drug differently than the considered medication, prescribing drug in different dose than required and prescribing different drug altogether than patient was already on e.g. Xalatan instead of Xalacom. Six (30%) patients were using their medicines at a wrong time or instilling a wrong dose and had difficulty in putting eye drops either because of bottle size, shape and type. One (5%) patient was given the wrong medicine by the pharmacy. During our study, eight (72.7%) patients experienced local side effects and three (27.3%) patients experienced systemic side effects. Of the local side effects, allergic reaction to preservatives was the most commonly observed side effect. A few patients reported hyperemia due to use of topical anti glaucoma medication. We prescribed preservative free drops in patients with allergic reactions to preservatives. Two patients experienced systemic side effects (from oral acetazolamide and topical beta blocker (Timolol).

Prescription errors committed by ophthalmologists were the most common error recorded during our study. Previous studies by Flynn *et al*, estimated the accuracy of dispensing prescriptions to be around 98.3%, with 6.5% of the errors judged to be clinically significant.[5] Computer-based prescribing systems minimize the risk of mistakes resulting from illegible prescriptions but this requires a lot of investment and training.[2] As many patients requiring ocular medications have poor sight, mishaps due to erroneous identification of the bottles[2] and small and hard to squeeze bottles impose major problem in proper ocular drug delivery. We recommend that medications should be double checked at the time of dispensing not only by the physician but also by the pharmacy and nurses. Patients should be educated about the timing of drops and proper gap in between two drops. Patient with arthritis and weakness of the hands should be kept in mind and they should be offered auto droppers where possible. Side effects to topical medications may be prevented by taking more detailed history about allergies to drugs, especially preservatives.
